# Rice yield prediction using UAV-based multispectral imagery and AutoGluon across regions and field scales

**DOI:** 10.3389/fpls.2026.1866530

**Published:** 2026-07-08

**Authors:** Jing Huo, Qingsong Wang, Chengyang Ji, Song Huang, Boteng Sun, Huifang Xiao, Chenyao Yang, Fengjun Yan, Yuxuan Zhang, Wei Zhou

**Affiliations:** 1 College of Agronomy, Sichuan Agricultural University, Chengdu, China; 2 School of Agronomy and Horticulture, Chengdu Agricultural College, Chengdu, China; 3 College of Intelligent Science and Engineering, Beijing University of Agriculture, Beijing, China; 4 Department of Computer and Electrical Engineering, Mid Sweden University, Sundsvall, Sweden

**Keywords:** Precision agriculture, canopy texture, AutoGluon, spatial transferability, rice

## Abstract

**Introduction:**

Accurate and transferable rice yield prediction is essential for precision agriculture and food security, yet existing remote sensing–based models often suffer from limited generalization across regions, cultivars, and field scales.

**Methods:**

This study developed an interpretable automated machine learning framework for rice yield prediction using UAV-based multispectral imagery collected at the maturity stage. A total of 143 rice samples, including 79 experimental plots and 64 production fields across 15 counties in Sichuan Province, China, were investigated. 20 vegetation indices and 36 gray-level co-occurrence matrix texture features were extracted from multispectral orthomosaics, and three feature selection strategies: Pearson correlation coefficient (PCC), Random Forest feature importance (RF-I), and AutoGluon feature importance(AutoGluon-I), were systematically compared. Four regression approaches, including CatBoost, ExtraTrees, Random Forest, and an AutoGluon stacked ensemble, were evaluated using R^2^, RMSE, and MAE.

**Results:**

The results showed that the AutoGluon ensemble consistently outperformed individual machine learning models, improving testset R^2^ from 0.403–0.670 to 0.528–0.736. The best performance was achieved by combining Pearson correlation-based feature selection with AutoGluon, yielding a training R^2^ of 0.821 and a test R^2^ of 0.736, with RMSE and MAE values of 0.749 and 0.568 t ha^−1^, respectively. Shapley Additive Explanations (SHAP) analysis further revealed that texture features, particularly red-band contrast and angular second moment features, contributed substantially to yield prediction, indicating the importance of canopy structural heterogeneity at maturity.

**Discussion:**

Overall, the proposed PCC–AutoGluon–SHAP framework provides a lightweight, accurate, and interpretable approach for UAV-based rice yield estimation across heterogeneous field conditions, offering practical potential for scalable precision agriculture and regional yield monitoring.

## 1 Introduction

Rice, as a staple crop critical to global food security, plays a vital role in agricultural production (FAO et al., 2022). In recent years, rice yield growth has slowed due to increasingly frequent extreme weather events and aggravated pest and disease pressures, posing a growing threat to food security. Accurate yield prediction not only supports informed decision-making in rice production and ensures the stable operation of grain markets, but also serves as a key strategy for disaster prevention, mitigation, and securing food supply (Wan et al., 2020). Traditional yield estimation methods rely on destructive field sampling, which is labor-intensive, costly, and inefficient, failing to meet the demands of modern agriculture for timeliness and efficiency (Zhang et al., 2020; Wang et al., 2024). In contrast, remote sensing technologies have gained widespread application in rice yield prediction due to their non-destructive, rapid, and high-precision characteristics, and are emerging as a core approach for high-throughput agricultural monitoring (Sulik and Long, 2016; Wan et al., 2020).

Satellite remote sensing enables large-area coverage; however, its relatively low spatial resolution and long revisit intervals limit its effectiveness for yield prediction at the scale of smallholder fields (Wan et al., 2020; Weiss et al., 2020; Htun et al., 2023). In contrast, Unmanned Aerial Vehicle (UAV) platforms offer high spatial and temporal resolution, operational flexibility, and low deployment costs, making them particularly suitable for crop growth monitoring and agronomic assessment across diverse field conditions and spatial scales (Ganeva et al., 2022). UAVs equipped with multispectral sensors can capture reflectance information across multiple wavelengths, thereby providing deep learning models with richer and more detailed input features (Zhao et al., 2013; Tang et al., 2025). Moreover, UAV-based data acquisition typically generates high-fidelity, targeted datasets that are easier to store, manage, and analyze compared to satellite big data, which is especially advantageous in agricultural scenarios that require rapid decision-making (Deng et al., 2018; Baio et al., 2019; Li et al., 2022).As crops approach maturity, physiological changes such as leaf yellowing, senescence, and panicle drooping become evident. These changes lead to reduced chlorophyll content, which in turn alters reflectance in the red-edge and near-infrared regions. As a result, vegetation indices (VIs) and texture features (TFVs) derived from UAV multispectral imagery during the maturity stage tend to exhibit strong correlations with crop yield (Cheng et al., 2021). Significantly, while morphological variation may appear minimal in the final growth stage, the near-harvest period represents the stage where the 3D spatial heterogeneity of the canopy, induced by panicle maturity and drooping, is fully stabilized and finalized. From the perspective of practical deployment, such as agricultural insurance verification or governmental yield audits, monitoring at this finalized stage provides the most direct proxy for the actual grain-in-bin yield and effectively minimizes prediction errors caused by unpredictable late-season disasters (e.g., late-stage lodging) that could occur after the grain-filling period. Zhou et al. (2017) extracted VIs from UAV-based multispectral imagery captured at different growth stages and employed multiple linear regression to estimate rice yield. Their findings indicated that yield predictions were more accurate from the late grain-filling to the maturity stage (Zhou et al., 2017). Similarly, Impollonia et al. (2024) developed machine learning (ML) models for rice yield estimation, reporting significantly higher predictive accuracy at the maturity stage (R² = 0.81) compared to the tillering stage (R² = 0.56) (Impollonia et al., 2024).

ML regression has been extensively employed in rice yield prediction due to its ability to capture complex nonlinear and nonparametric relationships between predictor variables and yield outcomes (van Klompenburg et al., 2020; Cao et al., 2021; Lingwal et al., 2024; Zhou et al., 2025). However, conventional ML workflows, encompassing feature engineering, algorithm selection, and hyperparameter tuning, are often time-consuming and require substantial domain expertise (Ferreira et al., 2021). To address these limitations, several automated machine learning (AutoML) frameworks, such as Auto-Sklearn, AutoGluon, and H2O (LeDell and Poirier, 2020), have been proposed to streamline the development of high-performance predictive models.

Swain et al. (2025) systematically evaluated the performance of various AutoML tools in agricultural yield prediction and found that AutoGluon outperformed others in terms of accuracy and robustness (Swain et al., 2025). Gaber et al. (2024) further demonstrated that AutoGluon achieved superior spatial generalization in estimating gross primary productivity (GPP) by integrating remote sensing imagery with meteorological variables (Gaber et al., 2024). Similarly, Kheir et al. (2024) proposed a rapid crop yield estimation approach based on AutoML, which effectively combined multi-source data, including remote sensing, soil, and climate inputs, to produce cost-efficient, automated, and high-precision predictions across diverse crop types (Kheir et al., 2024). These findings collectively underscore the broad applicability and potential advantages of AutoGluon in agricultural yield modeling.

Although numerous studies have explored the feasibility of integrating UAV-based remote sensing with ML for rice yield prediction, several limitations remain. First, most existing models are developed based on small-scale experimental plots within a single region, making them less transferable to large agricultural areas with diverse environmental and management conditions (Huang et al., 2016; Jin et al., 2017). Second, conventional ML models often rely on manual hyperparameter tuning, which is time-consuming and susceptible to subjective bias, while the application of AutoML frameworks in rice yield prediction has not been extensively investigated. Third, many models fail to account for growth differences among rice cultivars, thereby limiting their stability and generalizability (Huang et al., 2015). To address these issues, this study employed data from 79 experimental plots and 64 production fields (totaling 21.324 ha) across multiple ecological sites in Sichuan Province. UAV-based multispectral imagery at the rice maturity stage and corresponding ground-truth yield measurements were collected. A combination of vegetation indices (VIs) and textural features (TFVs) was extracted and screened to develop yield prediction models using four ML algorithms, including AutoGluon, with a comprehensive evaluation of their predictive accuracy and cross-regional generalization performance.

The objectives of this study are as follows: (1) To develop robust yield prediction models applicable across diverse ecological zones and varying sample scales, and to systematically evaluate the stability and generalization capability of different algorithms; (2) To assess the advantages and limitations of various feature selection strategies and their influence on model performance and predictive accuracy, providing theoretical support for variable selection and precision agriculture applications; (3) To compare the performance of individual algorithms and comprehensively evaluate the applicability and benefits of the AutoGluon automated ensemble learning framework for rice yield prediction, quantifying the predictive potential of heterogeneous model and feature integration. The expected outcomes will provide low-cost and efficient tools for yield estimation in different rice-growing regions, supporting the implementation of remote sensing–based precision agriculture technologies through data and methodological contributions.

## 2 Materials and methods

### 2.1 Research area

**Figure 1:**
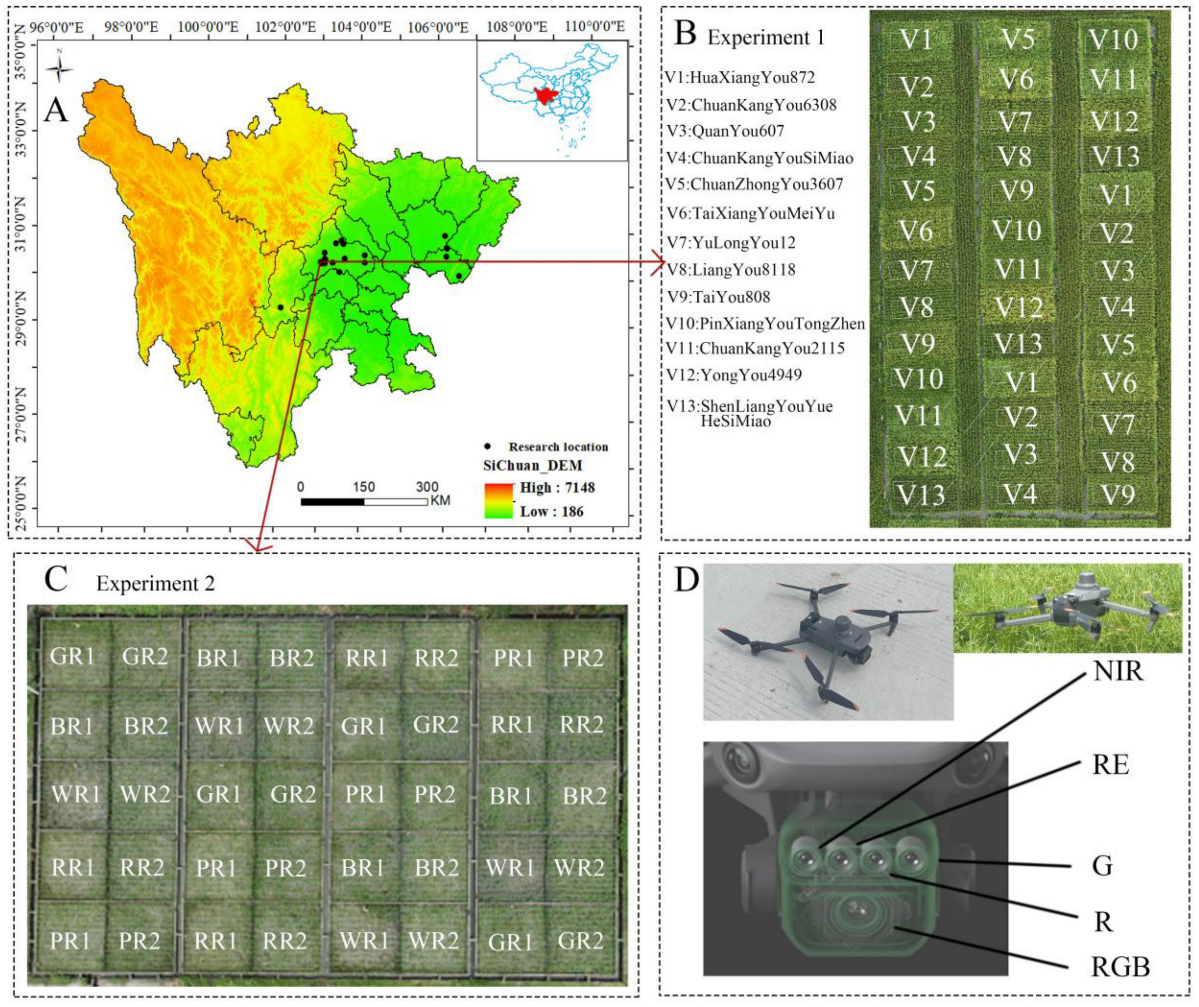
Data collection sites and schematic layout of plot experiments. **(A)** Distribution map of data collection points; **(B)** Field layout and variety distribution for Plot Experiment 1; **(C)** Field layout for Plot Experiment 2, illustrating five crop rotation patterns: Bean–Rice (BR), Rapeseed–Rice (RR), Wheat–Rice (WR), Potato–Rice (PR), and Garlic–Rice (GR), along with two management levels: Green Efficient Management (1) and Traditional Management (2); **(D)** UAV system and integrated sensor equipment, Near-infrared (NIR), RedEdge (RE), Green (G), Red (R).

This study was conducted between August 6 and September 26, 2024, across 15 counties in Sichuan Province, China. The extended data acquisition period resulted from the asynchronous maturity of rice across different regions. To ensure data quality and phenological consistency, the harvest timing for each plot was strictly determined by a panel of agronomic experts based on harvestable standards. Consequently, UAV multispectral imagery was acquired individually for each plot within 0–1 days prior to its actual harvest. A total of 143 highly heterogeneous rice field plots were investigated to obtain UAV-based multispectral imagery and corresponding ground-measured yield data. These plots encompassed varying field scales and management regimes, consisting of 64 large fields and 79 experimental plots (**Figure 1A**). The dataset deliberately combines homogeneously managed experimental plots with these large-scale commercial fields to overcome the limitations of idealized research environments. The inclusion of commercial fields introduces real-world spatial heterogeneity (e.g., uneven fertilization and edge effects), which are irreplaceable for evaluating the model’s cross-regional transferability and preventing overfitting. The large fields ranged from 0.013 to 1.068 hectares in area, totaling 21.324 hectares, all managed using mechanized transplanting and local high-yield cultivation practices.

The experimental plots comprised two distinct trials: (1) Experiment 1 (**Figure 1B**) was a rice varietal comparison trial conducted in Anren Town, Dayi County, Chengdu City, Sichuan Province. Images were acquired on September 18. The experiment involved 13 rice cultivars with 3 replicates each, totaling 39 plots. All plots were managed under mechanized transplanting, with each plot measuring 23.4 m²; (2) Experiment 2 (**Figure 1C**) was a long-term paddy-upland crop rotation experiment conducted at the Modern Agriculture Research and Development Base of Sichuan Agricultural University, Sichuan Province, with ‘ChuanKangYou 2115’ as the rice variety. Images were acquired on September 4. This trial employed manual transplanting, comprising 40 plots with an area of 16 m² each. The diverse spatial scale and ecological representation of these datasets provide a robust foundation for subsequent rice yield modeling and analysis.

### 2.2 Rice yield measurement

For the large-scale paddy fields across different ecological zones, rice yield was determined by full-area mechanical harvesting and weighing. Following the national expert acceptance protocol, five representative samples were randomly selected to determine impurity rate, using a grain moisture rapid tester to measure grain moisture content. The actual harvested area was determined using a land meter to calculate the final yield. For the experimental plots, rice was manually harvested, and each plot was processed individually. A 1-kg subsample was collected from each plot to determine impurity and moisture contents. The rice yield (Y) was then calculated using Equation 1, yield determination followed the standard method described by the Ministry of Agriculture of China (MOA, 2008), as widely used in previous studies:

(1) Y=W×(1−I)×(1−M)/(1−13.5%)/A×0.001

Where: Y = Actual yield (t·ha^−1^), W = Total grain weight (kg), I = Impurity rate, M = Moisture content, A = Harvested area (ha).

### 2.3 UAV-based multispectral data acquisition and preprocessing

#### 2.3.1 Multispectral image acquisition

To ensure image quality and comparability across geographically separated production fields and varying harvest dates, a rigorous, standardized quality control protocol was implemented across all sites. A DJI Mavic 3 Multispectral UAV (M3M; **Figure 1D**; technical specifications listed in **Supplementary Table S1**) was employed to acquire multispectral images of paddy fields under varying spatial scales and complex environmental conditions using a map-based flight mode. The UAV operated at an identical altitude of 20 m with a flight speed of 1.7 m/s. The longitudinal and lateral overlap ratios were uniformly set to 80% and 70%, respectively, with the camera oriented vertically at 90°. Furthermore, to eliminate radiometric variations caused by different flight dates and geographic locations, and to facilitate subsequent radiometric calibration of the remote sensing imagery and convert digital number (DN) values into surface reflectance, radiometric calibration panels (150 mm × 150 mm, CH-MXDJ) with known reflectance values of 25%, 50%, and 75% were placed within the study area prior to each flight. Since mechanized harvesting operations inherently require dry and clear weather, synchronizing UAV flights with the specific harvest schedule for each plot naturally ensured that all image acquisition missions were conducted under favorable, clear, and windless conditions. This alignment guaranteed sufficient and stable solar irradiance, flight safety, and gimbal stability, thereby maintaining high radiometric fidelity across the entire geographically dispersed dataset.

#### 2.3.2 Image processing

Multispectral images containing three calibrated reflectance reference panels and rice canopies were imported into Agisoft Metashape Professional 2.0.0 for radiometric correction, aiming to eliminate the influence of ambient illumination on image quality. Subsequently, the following photogrammetric workflow was executed within the software: image alignment, camera optimization, point cloud generation, digital elevation model (DEM) construction, and orthomosaic generation. The resulting orthomosaic images were then imported into ENVI 5.3.1, where regions of interest (ROIs) corresponding to individual field plots were manually delineated and exported as ESRI Shapefile (.shp) format. Finally, batch clipping of the orthomosaic imagery was conducted using Python 3.9.2 based on the defined ROI boundaries.

### 2.4 Remote sensing feature extraction and selection

#### 2.4.1 Extraction of vegetation indices and texture feature variables

In this study, 20 vegetation indices (VIs) widely used in yield prediction were selected, as listed in **Table 1**. These indices were strategically chosen to capture diverse biophysical and physiological traits of rice during the critical near-harvest period. Specifically, the selection includes: (1) pigment-sensitive indices (e.g., MCARI, MTCI) to assess chlorophyll content and photosynthetic capacity; (2) structural indices (e.g., NDVI, RDVI) to characterize canopy density and biomass accumulation; and (3) red-edge-based indices (e.g., NDRE, REPle) which are highly sensitive to crop nitrogen status and early senescence, thus providing a more comprehensive feature space for robust yield estimation across heterogeneous scenarios. Additionally, nine commonly applied texture feature variables (TFVs) were derived based on the gray-level co-occurrence matrix (GLCM) (Haralick et al., 1973), including mean, variance, homogeneity, contrast, dissimilarity, entropy, correlation, angular second moment (ASM), and energy. Given that the M3M multispectral camera includes four single-band channels, a total of 36 TFVs were obtained. Each TFV was named according to the band and the corresponding texture metric (e.g., the mean of the green band is denoted as G_mean).

**Table 1:** Vegetation indices and calculation formulas.

Serial number	Name	Equation	Reference
1	Green Chlorophyll Index	$\text{Clg}=\frac{NIR}{G}-1$	(Gitelson and Merzlyak, 1994)
2	Red Edge Chlorophyll Index	$\text{Clre}=\frac{NIR}{RE}-1$	(Gitelson et al., 2003)
3	Double Area Triangle Transformation	$\text{DATT}=\frac{NIR-RE}{NIR-R}$	(Datt, 1999)
4	Modified Enhanced Vegetation Index	$\text{M}*\text{EVI}=\frac{2.5\left(NIR-R\right)}{NIR+6R-7.5G+1}$	(Gaw, 2018)
5	Green Normalized Difference Vegetation Index	$\text{GNDVI}=\frac{NIR-G}{NIR+G}$	(Gitelson et al., 1996)
6	Green-Red Vegetation Index	$\text{GRVI}=\frac{G-R}{G+R}$	(Motohka et al., 2010)
7	Green Wide Dynamic Range Vegetation Index	$\text{GWDRVI}=\frac{0.3NIR-G}{0.3NIR+G}+\frac{1-0.3}{1+0.3}$	(Zhang et al., 2015)
8	Modified Chlorophyll Absorption in Reflectance Index	$\text{MCARI}=\frac{RE\left(RE-R-0.2\left(RE-G\right)\right)}{R}$	(Daughtry et al., 2000)
9	Modified Simple Ratio	$\text{MSR}=\frac{(NIR/R)-1}{\sqrt{(NIR/R)}+1}$	(Chen, 1996)
10	Meris Terrestrial Chlorophyll Index	MTCI=(NIR−RE)/(RE−R)	(Dash and Curran, 2004)
11	Modified Triangular Vegetation Index 2	$\text{MTVI}2=\frac{1.5\left(1.2\left(NIR-G\right)-2.5\left(R-G\right)\right)}{\sqrt{{\left(2NIR+1\right)}^{2}-\left(6NIR-5\sqrt{R}\right)}-0.5}$	(Lausch et al., 2013)
12	Normalized Difference Red Edge	$\text{NDRE}=\frac{NIR-RE}{NIR+RE}$	(Jiang et al., 2020)
13	Normalized Difference Vegetation Index	$\text{NDVI}=\frac{NIR-R}{NIR+R}$	(Rouse et al., 1974)
14	Canopy Chlorophyll Content Index	$\text{CCCI}=\frac{NDRE}{NDVI}$	(Barnes et al., 2000)
15	Optimization of Soil-Adjusted Vegetation Index	$\text{OSAVI}=\frac{1.16\left(NIR-R\right)}{NIR+R+0.16}$	(Rondeaux et al., 1996)
16	Renormalized Difference Vegetation Index	$\text{RDVI}=\frac{NIR-R}{\sqrt{NIR+R}}$	(Roujean and Breon, 1995)
17	Red Edge Position linear interpolation	$\text{REPIe}=700+40\frac{\left(R+RE\right)/2-RE}{RE+R}$	(Guyot and Baret, 1988)
18	Red Edge Ratio Vegetation Index	$\text{RERVI}=\frac{NIR}{RE}$	(Barzin et al., 2022)
19	Triangular Vegetation Index	$\text{TVI}=\frac{120\left(NIR-G\right)-200\left(R-G\right)}{2}$	(Broge and Leblanc, 2001)
20	Optimal Vegetation Index	$\text{Vlopt}=\frac{1.45\left(NIR^{2}+1\right)}{R+0.45}$	(Reyniers et al., 2006)

Where R denotes the red band, G the green band, RE the red-edge band, and NIR the near-infrared band.

To enable accurate yield prediction of rice across fields of varying scales and ecological zones in Sichuan Province using UAV-based multispectral imagery, an automated Python-based workflow was developed. This pipeline was employed to batch-extract spectral vegetation indices (VIs) and texture feature variables (TFVs) from all pre-processed multispectral images. The automation significantly improved both the efficiency and consistency of feature extraction, ensuring reproducibility and precision in the analysis of large-scale, multi-temporal remote sensing data.

#### 2.4.2 Selection of remote sensing features

**Figure 2:**
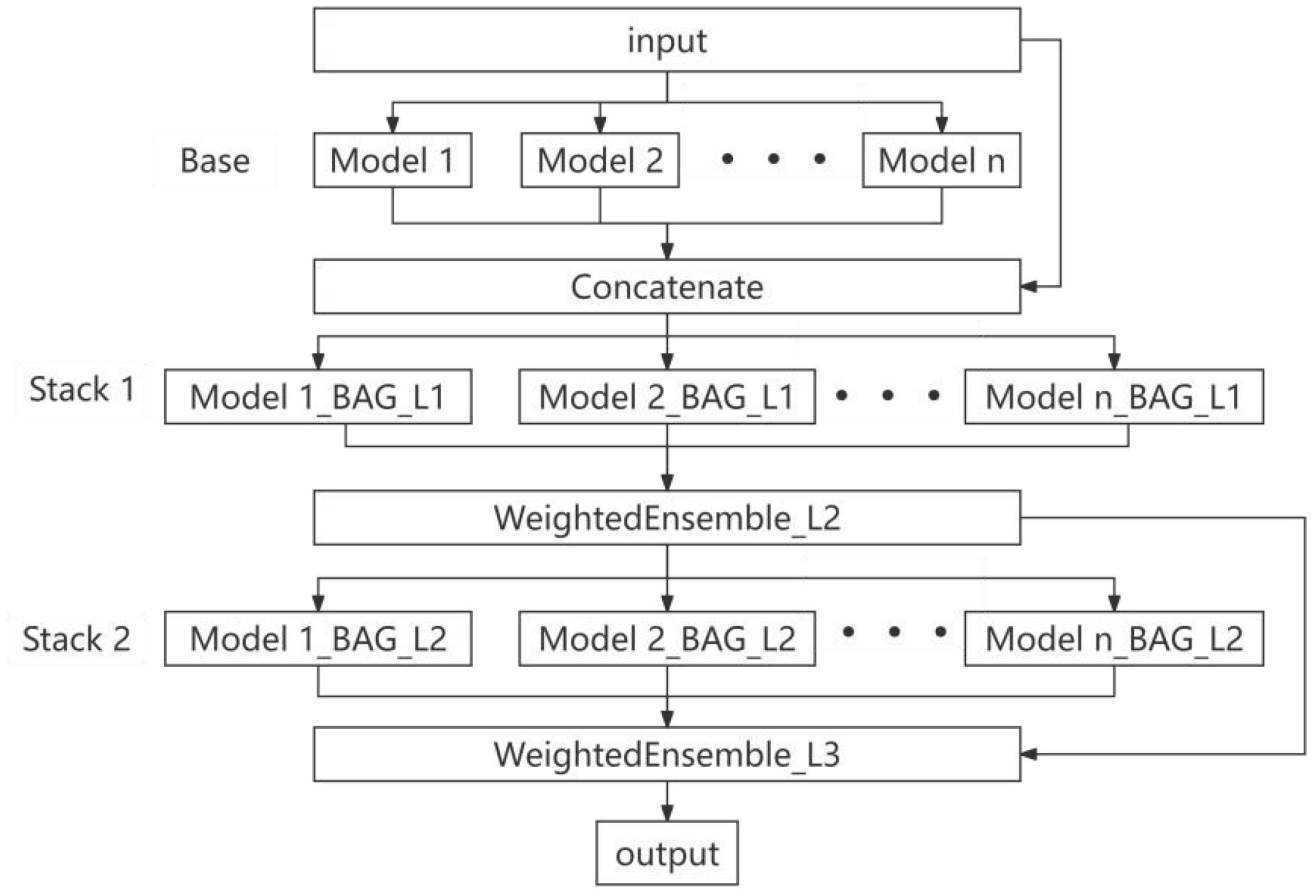
Multilayer stacking strategy of AutoGluon (Swain et al., 2025).

To reduce model complexity, mitigate the risk of overfitting, and enhance prediction performance, it is essential to conduct effective feature selection prior to model construction, aiming to eliminate redundant, noisy, and irrelevant variables (Hajgude and Sarode, 2024). To comprehensively evaluate the impact of different feature selection strategies on model performance, three approaches were employed in this study: Pearson correlation coefficient (a typical filter-based method, PCC) (Pearson, 1895), random forest feature importance evaluation (a typical wrapper-based method, RF-I) (Breiman, 2001a), and automated feature importance assessment using AutoGluon (AutoGluon-I). AutoGluon-I is a hybrid embedded + wrapper feature selection method that leverages a built-in stacking ensemble framework (**Figure 2**) to jointly train multiple heterogeneous models. By aggregating feature importance scores across models via a weighted voting strategy, it provides a robust estimation of feature relevance (Erickson et al., 2020). This method operates without the need for manual hyperparameter tuning, making it particularly suitable for scenarios involving a large number of features and high model complexity.

In this study, the top-performing algorithms within the AutoGluon framework were CatBoost, Random Forest (RF), and ExtraTrees (ET). To improve computational efficiency and manage resource consumption, subsequent analyses using AutoGluon were restricted to the stacking ensemble of these three models. Therefore, all references to “AutoGluon” hereafter denote the automatic stacked model based on the CatBoost + ET + RF ensemble within the AutoGluon framework.

### 2.5 Model development and evaluation

#### 2.5.1 Model development

Based on Python 3.9.2, four categories of ML algorithms were selected to develop rice yield prediction models: (1) CatBoost (Prokhorenkova et al., 2018); (2) RF (Breiman, 2001a); (3) ET (Geurts et al., 2006a); and (4) AutoGluon automated stacked ensemble model. The latter integrates the predictive advantages of the aforementioned base learners through multi-level stacking and dynamic weight optimization (Erickson et al., 2020). This ensemble framework supports automated hyperparameter tuning and feature engineering, substantially reducing the need for manual intervention.

To ensure scientific rigor and stability in model evaluation, as well as to enhance model generalization, CatBoost, RF, and ET models employed 10-fold cross-validation (K-Fold = 10) combined with Bayesian optimization for optimal hyperparameter search (Snoek et al., 2012). AutoGluon utilized its built-in automated settings (num_bag_folds = 10) to control the number of internal cross-validation folds and defined hyperparameter spaces for tuning diverse base models. This mechanism completes model fusion and optimal parameter search without human intervention, demonstrating the high adaptability and efficiency of the automated modeling framework in complex agricultural remote sensing prediction tasks.

#### 2.5.2 Model evaluation

**Figure 3:**
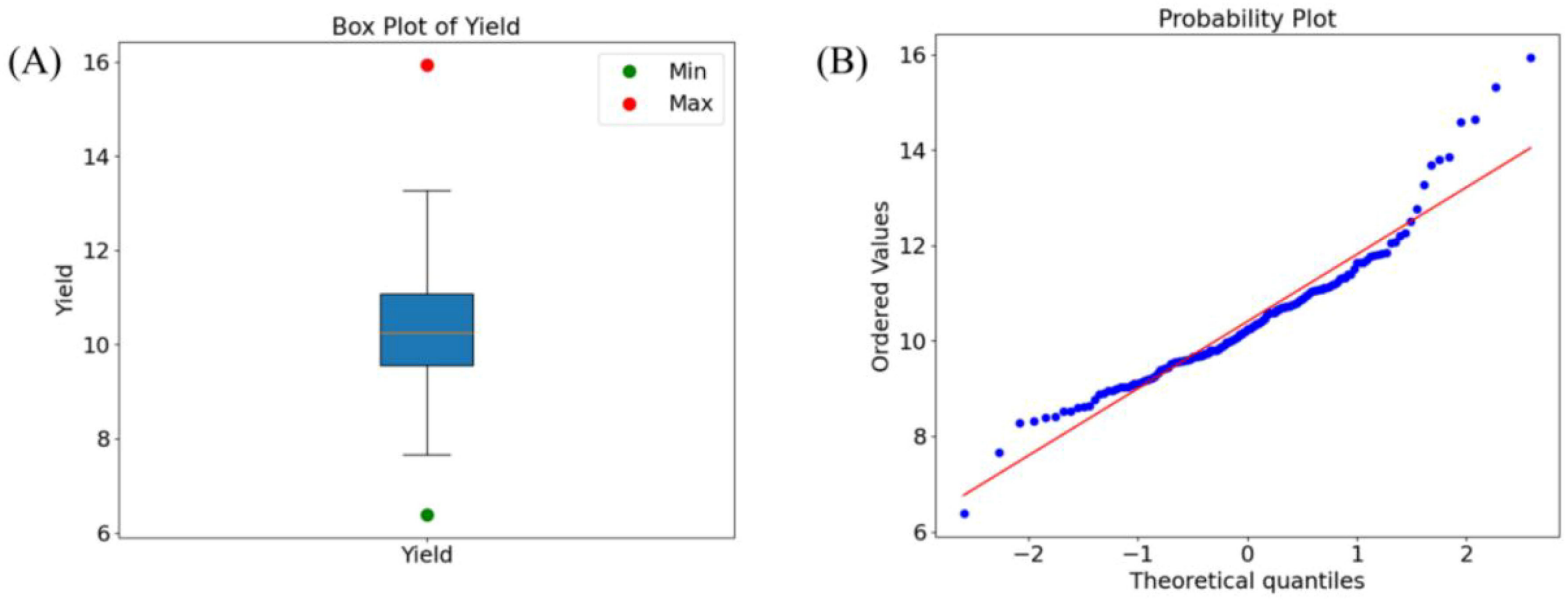
Visualization of yield distribution. **(A)** Boxplot of yield; **(B)** Quantile–Quantile (Q–Q) plot of yield.

In this study, distributional visualization of the yield dataset was performed (**Figure 3**; **Supplementary Figure S1**) to identify and exclude two extreme outliers corresponding to the maximum (15.94 t/ha) and minimum (6.38 t/ha) yield values (Tukey, 1977). Consequently, a total of 141 samples were retained for modeling analysis. The dataset was randomly split into training and testing sets at a ratio of 4:1, with 113 samples used for training to fit the models and 28 samples reserved for evaluating predictive performance. Model stability and reliability were assessed using the coefficient of determination (R²), root mean square error (RMSE), and mean absolute error (MAE). It should be noted that R² is a dimensionless metric representing the proportion of variance explained by the model, whereas both RMSE and MAE are expressed in the same units as the target variable (i.e., t/ha), directly quantifying the absolute prediction error. These metrics were calculated as follows:

(2) $$R^{2}=1-\frac{\sum_{i=1}^{n}{\left(yi-\hat{y}i\right)}^{2}}{\sum_{i=1}^{n}{\left(yi-\bar{y}\right)}^{2}}$$

(3) $$RMSE=\sqrt{\frac{\sum_{i=1}^{n}{\left(yi-\hat{y}i\right)}^{2}}{n}}$$

(4) $$MAE=\frac{\sum_{i=1}^{n}\left|yi-\hat{y}i\right|}{n}$$

In Equations 2–4, *y_i_* denotes the observed yield value, $\hat{y}i$ represents the predicted yield value, $\bar{\text{y}}$ is the mean of the observed yield values, and *n* indicates the total number of yield samples.

#### 2.5.3 Model interpretation based on SHAP

To address the “black-box” nature of machine learning models and elucidate the underlying agronomic logic between remote sensing features and rice yield, this study employed the SHapley Additive exPlanations (SHAP) method based on cooperative game theory. SHAP quantifies the contribution of each input variable by calculating its marginal contribution (Shapley value), thereby achieving a unified framework for both global and local model explanations. The core principle of SHAP is to decompose the predicted output into a linear sum of individual feature contributions (Equation 5):

(5) $$\text{y}=\phi_{0}+\sum_{j=1}^{M}\phi_{j}$$

Where y represents the predicted yield; ϕ_0_ is the base value (the average predicted output across the training dataset); ϕ_j_ is the SHAP value for the j-th feature, representing the quantified measure of how much that feature drives the prediction to deviate from the base value; and M is the total number of input features.

## 3 Result

### 3.1 Distribution analysis of rice yield

**Figure 4:**
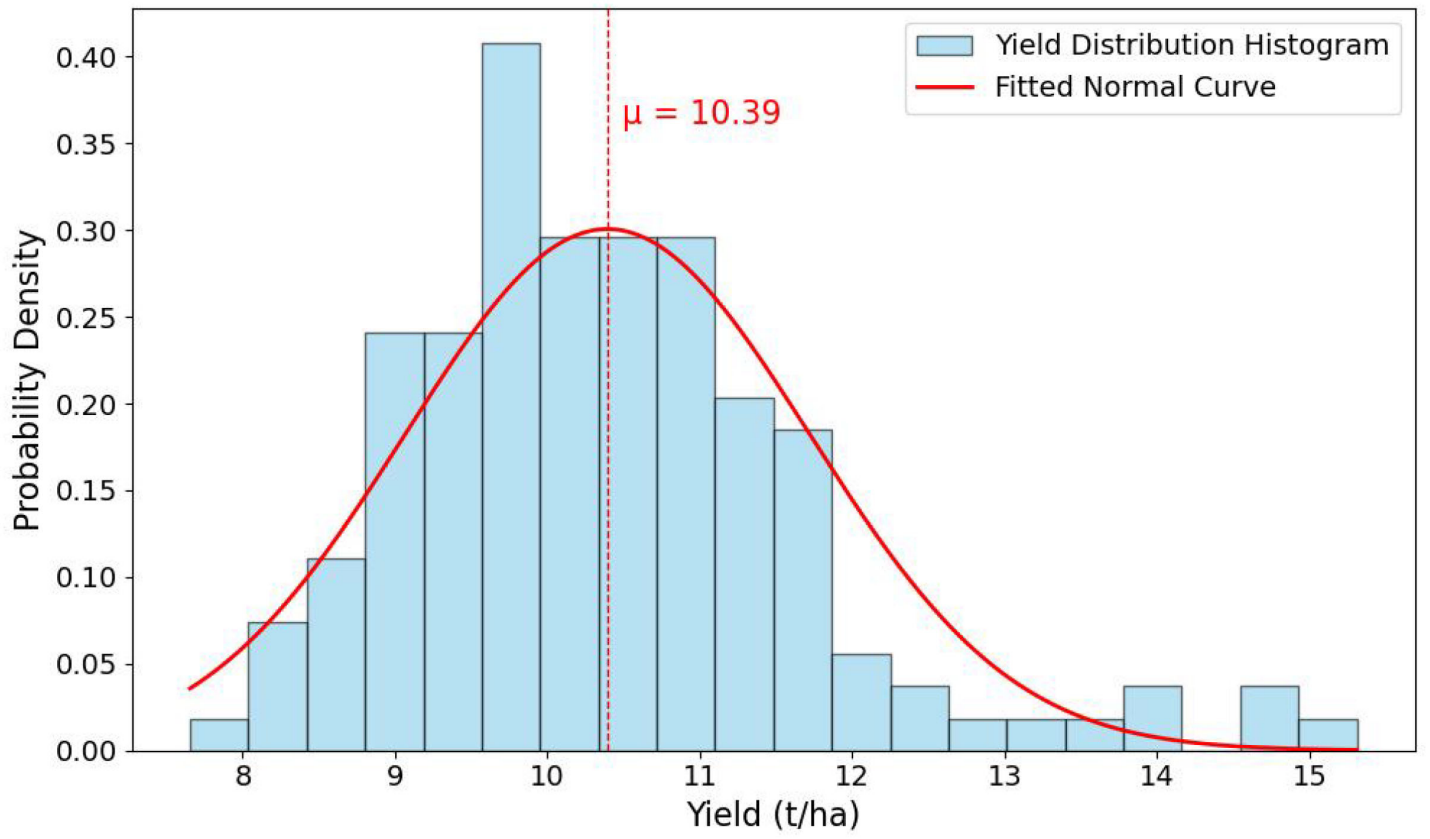
Probability density distribution and normal fitting curve of rice yield. The figure illustrates the kernel density estimation of yield values overlaid with a normal distribution curve, enabling evaluation of the distribution characteristics and testing the assumption of normality for the yield variable.

As shown in **Figure 4**, the rice yield distribution in this dataset encompasses low-, medium-, and high-yield levels, indicating strong representativeness. The yield data exhibit an approximately normal distribution, with a mean (μ) of approximately 10.39 t/ha. Although slightly skewed toward the high-yield end, both skewness and kurtosis remain close to those of a standard normal distribution, supporting the dataset’s suitability for the development of robust yield prediction models.

### 3.2 Optimal feature selection under different algorithms

**Figure 5:**
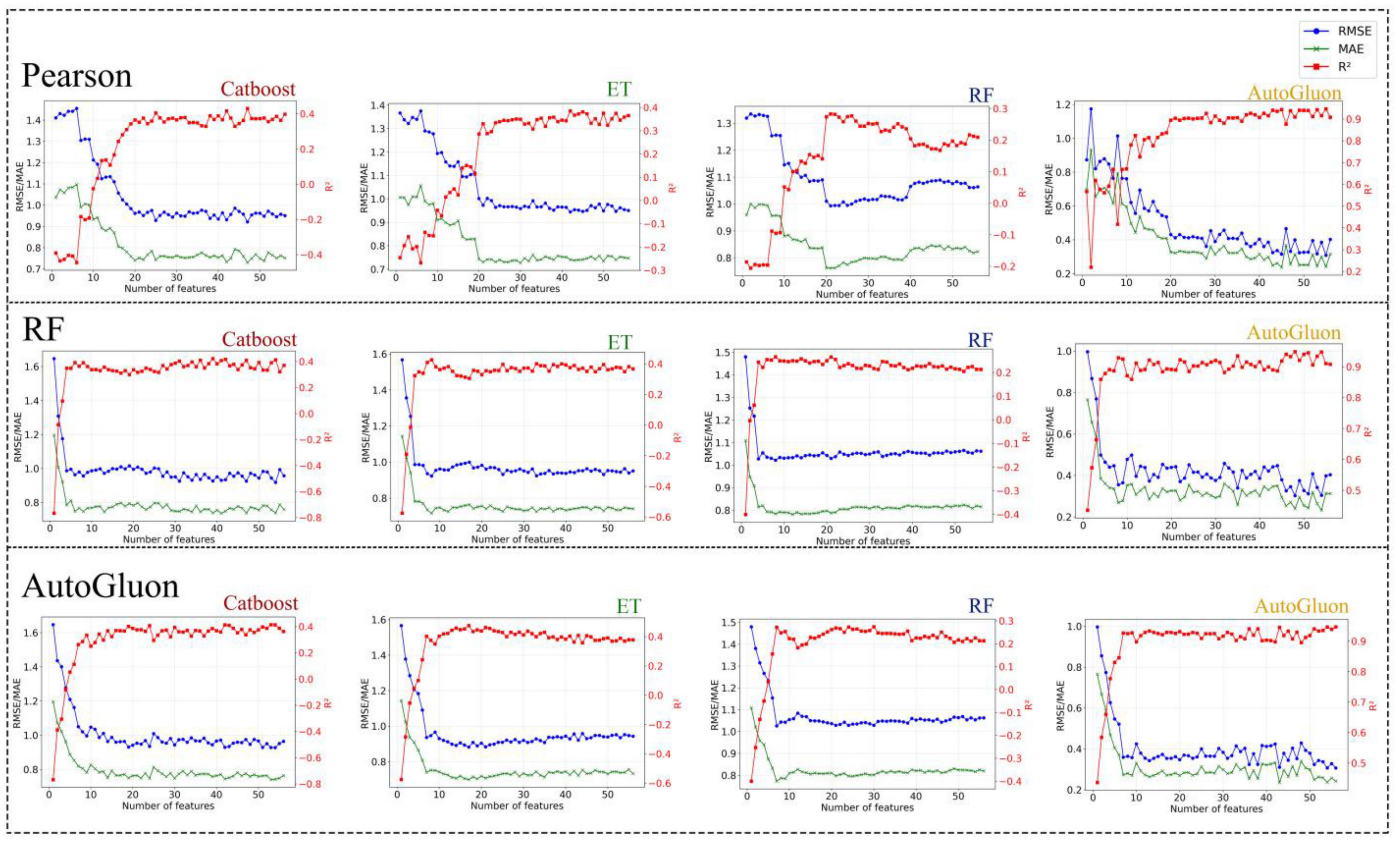
Modeling performance and inflection point identification of different ML algorithms under various feature selection strategies.

Based on the feature importance rankings derived from three selection strategies (PCC, RF-I, and AutoGluon-I; **Supplementary Tables S2–S4**), subsets with increasing numbers of top-ranked features were progressively constructed. Subsequently, four ML algorithms: CatBoost, ET, RF, and AutoGluon, were employed to model rice yield using these subsets. For each algorithm and feature selection method, the performance trends (in terms of R², RMSE, and MAE) were plotted as functions of feature count (**Figure 5**), enabling the identification of performance inflection points.

Results indicate that although the optimal number of features varies among selection strategies and algorithms, a general trend is observed: model accuracy improves with increasing feature count until reaching a plateau. Specifically, the PCC and RF-I methods yielded identical optimal feature counts across the four models, with 20 and 8 features, respectively. In contrast, AutoGluon-I ranked features led to 9, 7, 7, and 7 selected variables for the respective models. These findings underscore the utility of integrating feature importance rankings with incremental subset construction in identifying the critical number of variables that substantially contribute to model performance.

Under low-dimensional conditions (≤20 features), the performance curves of models based on Pearson correlation coefficient (PCC) exhibit notable fluctuations. This indicates that feature subsets ranked solely by linear correlation initially lack coverage of high-order interaction signals, rendering the models more sensitive to newly added weakly correlated variables. As additional secondary features are introduced, these interaction effects are gradually complemented, leading to a performance inflection point around 20 features and subsequent stabilization. This feature selection strategy not only efficiently captures the primary linear driving factors but also facilitates the extraction of high-order interactions upon inclusion of a moderate number of secondary variables, thereby enhancing the overall predictive performance.

### 3.3 Performance of single-algorithm-based rice yield prediction models

**Figure 6:**
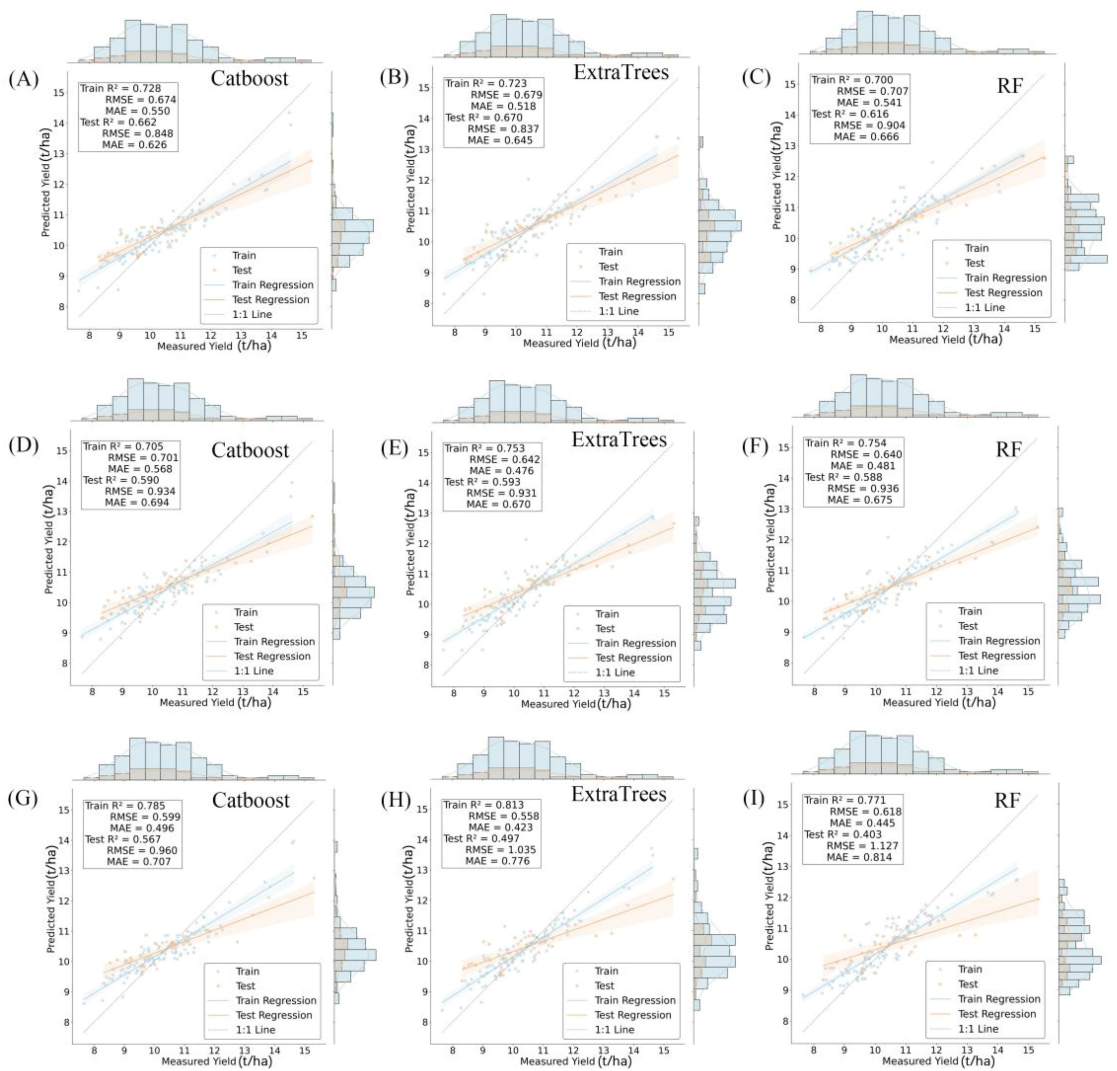
Performance of yield prediction models constructed using three individual ML algorithms (CatBoost, ET, and RF) based on three different feature selection strategies: PCC **(A–C)**, RF-I **(D–F)**, and AutoGluon-I **(G–I)**, evaluated on both training and testing sets.

Single-model yield prediction frameworks were developed using three ML algorithms (CatBoost, ET, and RF) combined with three different feature selection strategies (**Figure 6**). Results demonstrated that, on the training set, all three algorithms exhibited strong fitting capabilities under most feature selection schemes. The predicted values were highly linearly correlated with the observed yields, with regression lines nearly coinciding with the 1:1 reference line, indicating the models’ ability to capture patterns embedded in the training data. In contrast, the generalization performance on the test set revealed clear discrepancies across feature selection methods. Models utilizing feature subsets derived from Pearson correlation coefficient (PCC) consistently outperformed others across all three algorithms, achieving R^2^ values between 0.612 and 0.670, along with the lowest RMSE and MAE scores. Conversely, models built upon the AutoGluon-I-based feature selection performed the poorest on the test set, with R^2^ values ranging from 0.403 to 0.567.

These findings suggest that although single algorithms can achieve strong in-sample fitting, their robustness and predictive ability may be limited when handling complex feature interactions and non-linear relationships. While AutoGluon-I demonstrates the potential for feature dimensionality reduction, its feature ranking in this study might have excluded variables critical for accurate yield prediction, thereby compromising model generalization on unseen data.

### 3.4 Performance of rice yield prediction model based on AutoGluon

**Figure 7:**
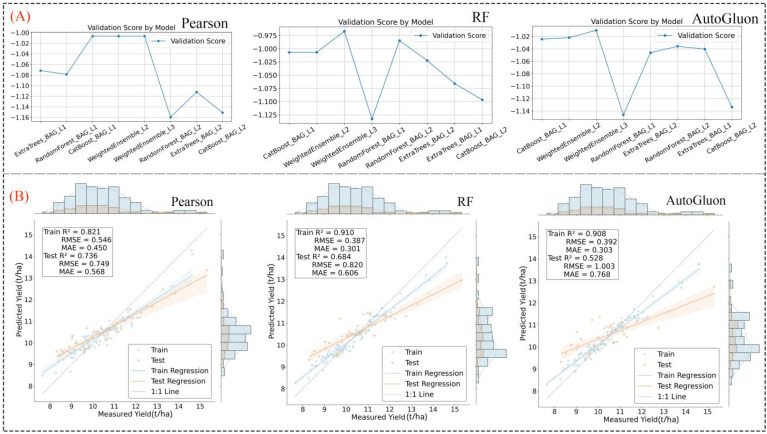
Visualization of learning curves **(A)** and model performance on the training and testing sets **(B)** of yield prediction models constructed using AutoGluon under three feature selection strategies (PCC, RF-I, and AutoGluon-I).

Based on the optimal combination of feature variables, a rice yield prediction model was constructed using the AutoGluon AutoML framework. The final prediction was uniformly derived from its top-level stacked ensemble output (weighted_ensemble_L3) (**Figure 7A**). It should be noted that the internal validation score used to evaluate model performance during the ensembling process was based on the Root Mean Squared Error (RMSE), expressed in units of tons per hectare (t/ha). To align with the maximization optimization logic of the framework, these scores are inherently represented as negative values. The results indicated that the model exhibited strong fitting capabilities under all three feature selection strategies. AutoGluon consistently outperformed individual base models (**Figures 6**, **7B**), maintaining high predictive accuracy and generalization ability across different feature selection methods.

Notably, the combination of PCC and AutoGluon achieved the best overall performance, with a testing R² of 0.736, RMSE reduced to 0.749, and MAE to 0.568—substantially better than other feature selection approaches. These findings confirm the feasibility and effectiveness of integrating a linear feature ranking strategy with a robust automated modeling system. In contrast, while AutoGluon-I demonstrates the potential for feature dimensionality reduction, its feature ranking in this study might have excluded variables critical for accurate yield prediction, thereby compromising model generalization on unseen data.

**Figure 8:**
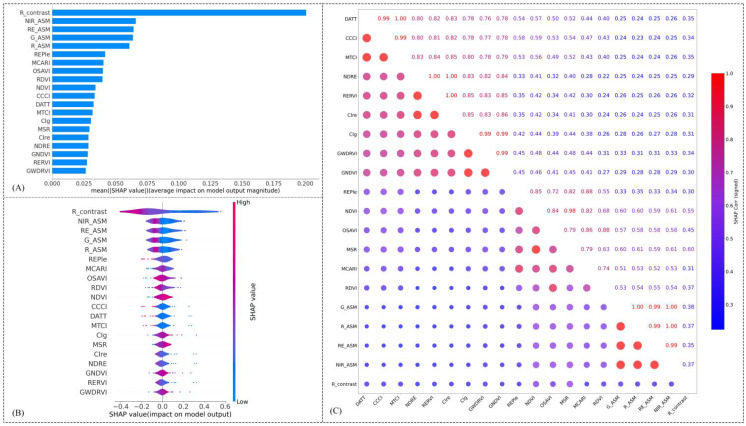
SHAP-based global feature contribution and interaction analysis. **(A)** Summary bar plot ranking the top features according to the mean absolute SHAP values, indicating their global importance to the prediction model. **(B)** SHAP beeswarm plot visualizing the distribution of SHAP values for individual samples across major features; each point represents a sample, with color indicating the feature value and position indicating the contribution to the predicted yield. **(C)** SHAP interaction bubble plot revealing the pairwise interaction effects between features, with bubble size reflecting the strength of the interaction and color denoting the direction and magnitude of the combined contribution to the model output.

To further enhance the interpretability of the model, a global SHAP analysis was conducted on the feature set selected by the PCC + AutoGluon combination (**Figure 8**). The results demonstrated that R_contrast emerged as the key driving variable, exhibiting the highest mean absolute SHAP value (mean(|SHAP|) ≈ 0.20), thereby contributing most significantly to the yield prediction model. The wide distribution of SHAP values along the horizontal axis for this feature (**Figure 8b**) indicates a high degree of individual variability or potential nonlinear interactions with other features. Although RERVI and GWDRVI individually showed relatively weak contributions (mean(|SHAP|)< 0.0375), their interactions with other vegetation indices substantially enhanced their explanatory power, as illustrated in **Figure 8c**. The SHAP interaction analysis further revealed strong synergistic effects between specific feature pairs, such as DATT–CCCI and NDRE–RERVI, with interaction values exceeding 0.8. These findings underscore the critical role of multi-feature coupling mechanisms in accurately modeling rice yield. In summary, this section unveils the model’s dependence on key remote sensing features from both global and interaction-based perspectives, providing a theoretical basis for feature refinement and model simplification in subsequent studies.

## 4 Discussion

### 4.1 Comparative performance analysis of AutoGluon under different feature selection strategies

Feature selection is pivotal for constructing high-performing ML models. In this study, the PCC-based method demonstrated superior performance over the AutoGluon-I strategy (**Figures 6**, **7**). By directly capturing dominant linear driving forces, PCC effectively retained core predictive information (20 features, **Supplementary Figure S2**) while mitigating the risk of underfitting caused by information deficiency (Whitmire et al., 2021; Li et al., 2023). In contrast, AutoGluon-I’s aggressive pruning, retaining only 7–9 features based on ensemble marginal gains, often lacked sufficient interaction support when modeled by individual algorithms, ultimately resulting in performance degradation (Erickson et al., 2020; Chatzimparmpas et al., 2021). The PCC-selected set provided a robust foundation for AutoGluon’s built-in feature interaction generator to extract complex non-linear relationships, creating a complementary mechanism between linear screening and non-linear modeling.

Integrating PCC-selected features with the AutoGluon automated stacked ensemble (weightedensemble_L3) substantially improved the test set R² from a single-algorithm average of 0.649 to 0.736. Although CatBoost, ET, and RF each offer distinct advantages, their standalone performances were suboptimal in this study. The limited sample size (N = 143) and pronounced individual variability likely triggered overfitting in CatBoost’s ordered boosting and symmetric tree structures, compromising its generalization across regions (Prokhorenkova et al., 2018). Furthermore, the heterogeneous conditions of multiple varieties and ecological zones increased sensitivity to regional bias. Specifically, ET’s “extremely randomized” splitting and RF’s default column sampling often struggled to capture high-order interactions or discern interregional variability under complex environmental backgrounds, frequently resulting in high-variance outputs (Breiman, 2001a; Geurts et al., 2006a; Nicodemus et al., 2010).

Compared to single models, AutoGluon leverages multi-layered stacking and 10-fold bagging to generate robust out-of-fold predictions. This pipeline significantly reduces model variance and utilizes dynamic weight fusion to suppress the influence of localized noise, thereby mitigating overfitting in small-sample scenarios. Additionally, the framework’s built-in binning and interaction generators successfully transformed weakly linear predictors, such as R_contrast and NIR_ASM, into high-information-density features (**Supplementary Figure S2**) (Song et al., 2013; Chang et al., 2019). By automatically tuning hyperparameters and pruning underperforming models via the “best_quality” mode, AutoGluon prevented the error accumulation and model drift common in manual parameter tuning. In summary, the “PCC + AutoGluon” combination circumvented the limitations of standalone algorithms under complex agro-environmental conditions, significantly enhancing both fitting accuracy and cross-regional adaptability for rice yield estimation.

### 4.2 SHAP-based analysis of key feature contributions and response patterns

SHAP analysis was employed to quantify the marginal contributions of key features to the optimal model (Shapley, 1953). The results revealed that R_contrast and various band-specific ASM features (e.g., NIR_ASM, RE_ASM) played dominant roles, outperforming traditional VIs. Selected VIs fundamentally influence yield by characterizing physiological “sources” (e.g., MCARI for chlorophyll density) and biomass “accumulation” (e.g., NDVI for canopy density), which directly correlate with photosynthetic potential. However, VIs often encounter spectral saturation during the high-biomass maturity stage (Hao and Huang, 2023). Our findings highlight that TFVs effectively mitigate this limitation by capturing high-order structural information.

Notably, R_contrast exhibited the highest SHAP contribution by capturing the “interweaving intensity” between functional green leaves (Red-light absorbers) and maturing yellow panicles (Red-light reflectors). A high contrast value indicates a productive canopy where abundant “sinks” (panicles) are densely integrated with “sources” (leaves), serving as a robust proxy for panicle density and grain fullness. Furthermore, the consistently high SHAP values for ASM features (mean ≈ 0.10–0.12) emphasize the impact of canopy texture homogeneity on yield formation (Zheng et al., 2020), confirming that yield is driven more by canopy structure than narrow-band spectral responses at maturity.

These results align with previous studies where combining VIs and TFVs enhanced the inversion accuracy of LAI and dry matter (Zhang et al., 2021; Yuan et al., 2023). Nevertheless, the physiological causal link between TFVs and final yield remains complex. Future research incorporating empirical observations of leaf arrangement and light distribution is necessary to further validate these mechanistic relationships.

### 4.3 Limitations and future research

Despite the improved accuracy and interpretability provided by the PCC-AutoGluon framework, several constraints must be addressed to enhance its operational scalability.

One primary limitation lies in the spatiotemporal coverage of the training data. While the dataset encompasses significant variability in field scales (experimental *vs*. production) and cultivars, it is confined to a single climatic zone and phenological cycle. Consequently, the model’s generalization capability under extreme weather events or across distinct agro-ecological zones requires further validation. Extending the experimental design to include longitudinal data over multiple years is essential to assess the framework’s stability against temporal climate variability.

Furthermore, the current feature engineering approach focuses on instantaneous remote sensing observations. Although texture features significantly improved the representation of canopy heterogeneity, crop yield is a cumulative physiological response to gene-environment interactions. Relying solely on mature-stage data inherently entails a time lag, which limits its practical value for guiding early-season field management, such as timely topdressing or irrigation. In addition, the absence of meteorological data (e.g., thermal time, radiation) and soil properties limits the model’s capacity to explain yield variations driven by abiotic stresses. Future iterations of the framework should prioritize the integration of multi-temporal remote sensing datasets to enable early yield prediction and whole-growth-stage dynamic monitoring, while concurrently incorporate these environmental covariates to enhance biological relevance.

Finally, regarding spatial scalability, the reliance on UAV imagery constrains the direct deployment of the model to the plot or farm scale. To substantiate the framework’s applicability for large-scale precision agriculture, future work will investigate multi-source data fusion. Specifically, using UAV data as high-fidelity ground truth to calibrate satellite-based models (e.g., Sentinel-2) could leverage the efficiency of the proposed AutoGluon architecture for provincial-level yield estimation.

## 5 Conclusion

This study systematically evaluated the application of feature selection strategies based on UAV multispectral data and the AutoGluon automated modeling framework in rice yield prediction. The main findings are as follows:

1. Models constructed using features with strong linear correlations exhibited significantly superior predictive performance compared to other feature selection strategies, indicating that their simplicity effectively avoids noise interference from statistically complex but agronomically irrelevant features.
2. The AutoGluon automated stacked ensemble model achieved the best predictive performance (test set R² = 0.736), representing a 13% improvement over individual models (average R² = 0.649). This validates its capability to amplify subtle signals through heterogeneous model fusion (CatBoost, ET, RF) combined with automated feature engineering.
3. In the best-performing “PCC-AutoGluon” model, SHAP analysis revealed that texture feature variables (TFVs) contributed substantially more than vegetation indices (VIs) (e.g., R_contrast with mean |SHAP| ≈ 0.2), and the SHAP values of angular second moment across spectral bands were highly consistent, reflecting the importance and broad applicability of TFVs in rice yield prediction. The “PCC-AutoGluon-SHAP” framework provides a lightweight, highly predictive, and interpretable modeling paradigm for crop yield estimation.
4. In terms of policy implications, the automation and interpretability of this framework offer a viable pathway for scaling precision agriculture. It is recommended that agricultural authorities promote such cost-effective, transparency-driven monitoring tools to enhance regional yield statistics and provide objective data support for agricultural insurance. Ultimately, the continuous integration of multi-source environmental data into this framework will be key to establishing a robust, temporally generalizable decision support system for global food security.

## Data Availability

The raw data supporting the conclusions of this article will be made available by the authors, without undue reservation.

## References

[B1] Baio F. H. R. Silva E. Martins P. H. A. Junior C. S. Teodoro P. E. (2019). In situ remote sensing as a strategy to predict cotton seed yield. Biosci. J. 35, 1847–1854. doi: 10.14393/BJ-v35n6a2019-42261

[B2] Barnes E. M. Clarke T. R. Richards S. E. Colaizzi P. D. Haberland J. Kostrzewski M. . (2000). Coincident Detection of Crop Water Stress, Nitrogen Status, and Canopy Density Using Ground Based Multispectral Data (Madison, WI, USA: ASA–CSSA–SSSA).

[B3] Barzin R. Lotfi H. Varco J. J. Bora G. C. (2022). Machine learning in evaluating multispectral active canopy sensor for prediction of corn leaf nitrogen concentration and yield. Remote Sens. 14, 120. doi: 10.3390/rs14010120 30654563

[B4] Breiman L. (2001a). Random forests. Mach. Learn. 45, 5–32. doi: 10.1023/A:1010933404324 41886696

[B5] Broge N. H. Leblanc E. (2001). Comparing prediction power and stability of broadband and hyperspectral vegetation indices for estimation of green leaf area index and canopy chlorophyll density. Remote Sens. Environ. 76, 156–172. doi: 10.1016/S0034-4257(00)00197-8

[B6] Cao J. Zhang Z. Tao F. Zhang L. Luo Y. Zhang J. . (2021). Integrating multi-source data for rice yield prediction across China using machine learning and deep learning approaches. Agric. For. Meteorol. 297, 108275. doi: 10.1016/j.agrformet.2020.108275 38826717

[B7] Chang T. Zhao H. Wang N. Song Q. Xiao Y. Qu M. . (2019). A three-dimensional canopy photosynthesis model in rice with a complete description of the canopy architecture, leaf physiology, and mechanical properties. J. Exp. Bot. 70, 2479–2490. doi: 10.1093/jxb/ery430 30801123 PMC6487591

[B8] Chatzimparmpas A. Martins R. M. Kucher K. Kerren A. (2021). StackGenVis: Alignment of data, algorithms, and models for stacking ensemble learning using performance metrics. IEEE Trans. Visual Comput. Graphics 27, 1547–1557. doi: 10.1109/TVCG.2020.3030352 33048687

[B9] Chen J. M. (1996). Evaluation of vegetation indices and a modified simple ratio for boreal applications. Can. J. Remote Sens. 22, 229–242. doi: 10.1080/07038992.1996.10855178 37339054

[B10] Cheng Q. Xu H. Cao Y. Duan F. Chen Z. (2021). Grain yield prediction of winter wheat using multi-temporal UAV based on multispectral vegetation index [in Chinese. Trans. Chin. Soc Agric. Mach. 52, 160–167. doi: 10.6041/j.issn.1000-1298.2021.03.017

[B11] Dash J. Curran P. J. (2004). The MERIS terrestrial chlorophyll index. Int. J. Remote Sens. 25, 5403–5413. doi: 10.1080/0143116042000274015 37339054

[B12] Datt B. (1999). Visible/near infrared reflectance and chlorophyll content in eucalyptus leaves. Int. J. Remote Sens. 20, 2741–2759. doi: 10.1080/014311699211778 37339054

[B13] Daughtry C. S. T. Walthall C. L. Kim M. S. de Colstoun E. B. McMurtrey J. E. (2000). Estimating corn leaf chlorophyll concentration from leaf and canopy reflectance. Remote Sens. Environ. 74, 229–239. doi: 10.1016/S0034-4257(00)00113-9

[B14] Deng L. Mao Z. Li X. Hu Z. Duan F. Yan Y. (2018). UAV-based multispectral remote sensing for precision agriculture: A comparison between different cameras. ISPRS J. Photogramm. Remote Sens. 146, 124–136. doi: 10.1016/j.isprsjprs.2018.09.008 38826717

[B15] Erickson N. Mueller J. Shirkov A. Zhang H. Larroy P. Li M. . (2020). AutoGluon-Tabular: Robust and accurate AutoML for structured data. doi: 10.48550/arXiv.2003.06505

[B16] FAO IFAD UNICEF WFP WHO (2022). The State of Food Security and Nutrition in the World 2022:Repurposing Food and Agricultural Policies to Make Healthy Diets More Affordable (Rome, Italy: FAO).

[B17] Ferreira L. Pilastri A. Martins C. M. Pires P. M. Cortez P. (2021). A comparison of AutoML tools for machine learning, deep learning and XGBoost 1–8. doi: 10.1109/IJCNN52387.2021.9534091

[B18] Gaber M. Kang Y. Schurgers G. Keenan T. (2024). Using automated machine learning for the upscaling of gross primary productivity. Biogeosciences 21, 2447–2472. doi: 10.5194/bg-21-2447-2024

[B19] Ganeva D. Roumenina E. Dimitrov P. Gikov A. Jelev G. Dragov R. . (2022). Phenotypic traits estimation and preliminary yield assessment in different phenophases of wheat breeding experiment based on UAV multispectral images. Remote Sens. 14, 1019. doi: 10.3390/rs14041019 30654563

[B20] Gaw L. Y. F. (2018). Unmanned Aerial Systems in Remotely Sensed Biomass Estimates: How They Improve the Quality of Existing Satellite Based Approaches (Münster: University of Münster).

[B21] Geurts P. Ernst D. Wehenkel L. (2006a). Extremely randomized trees. Mach. Learn. 63, 3–42. doi: 10.1007/s10994-006-6226-1 30311153

[B22] Gitelson A. A. Gritz Y. Merzlyak M. N. (2003). Relationships between leaf chlorophyll content and spectral reflectance and algorithms for non-destructive chlorophyll assessment in higher plant leaves. J. Plant Physiol. 160, 271–282. doi: 10.1078/0176-1617-00887 12749084

[B23] Gitelson A. A. Kaufman Y. J. Merzlyak M. N. (1996). Use of a green channel in remote sensing of global vegetation from EOS-MODIS. Remote Sens. Environ. 58, 289–298. doi: 10.1016/S0034-4257(96)00072-7

[B24] Gitelson A. Merzlyak M. N. (1994). Spectral reflectance changes associated with autumn senescence of Aesculus hippocastanum L. and Acer platanoides L. leaves. Spectral features and relation to chlorophyll estimation. J. Plant Physiol. 143, 286–292. doi: 10.1016/S0176-1617(11)81633-0 29786478

[B25] Guyot G. Baret F. (1988). Utilisation De La Haute Resolution Spectrale Pour Suivre L’etat Des Couverts Vegetaux (Aussois: ASE).

[B26] Hajgude J. Sarode T. (2024). Analyzing the impact of feature selection on crop yield prediction. J. Comput. Commun. 12, 278–291. doi: 10.4236/jcc.2024.128017

[B27] Hao Q. Huang C. (2023). A review of forest aboveground biomass estimation based on remote sensing data. Chin. J. Plant Ecol. 47, 1356–1374. doi: 10.17521/cjpe.2023.0008

[B28] Haralick R. M. Shanmugam K. Dinstein I. (1973). Textural features for image classification. IEEE Trans. Syst. Man Cybern. SMC-3, 610–621. doi: 10.1109/TSMC.1973.4309314 25079929

[B29] Htun A. M. Shamsuzzoha M. Ahamed T. (2023). Rice yield prediction model using normalized vegetation and water indices from Sentinel-2A satellite imagery datasets. Asia-Pac. J. Reg. Sci. 7, 491–519. doi: 10.1007/s41685-023-00299-2 30311153

[B30] Huang J. Sedano F. Huang Y. Ma H. Li X. Liang S. . (2016). Assimilating a synthetic Kalman filter leaf area index series into the WOFOST model to improve regional winter wheat yield estimation. Agric. For. Meteorol. 216, 188–202. doi: 10.1016/j.agrformet.2015.10.013 38826717

[B31] Huang J. Tian L. Liang S. Ma H. Becker-Reshef I. Huang Y. . (2015). Improving winter wheat yield estimation by assimilation of the leaf area index from Landsat TM and MODIS data into the WOFOST model. Agric. For. Meteorol. 204, 106–121. doi: 10.1016/j.agrformet.2015.02.001 38826717

[B32] Impollonia G. Croci M. Amaducci S. (2024). Upscaling and downscaling approaches for early season rice yield prediction using Sentinel-2 and machine learning for precision nitrogen fertilisation. Comput. Electron. Agric. 227, 109603. doi: 10.1016/j.compag.2024.109603 38826717

[B33] Jiang J. Wang C. Wang Y. Cao Q. Tian Y. Zhu Y. . (2020). Using an active sensor to develop new critical nitrogen dilution curve for winter wheat. Sens. 20, 1577. doi: 10.3390/s20061577 32178244 PMC7146448

[B34] Jin X. Li Z. Yang G. Yang H. Feng H. Xu X. . (2017). Winter wheat yield estimation based on multi-source medium resolution optical and radar imaging data and the AquaCrop model using the particle swarm optimization algorithm. ISPRS J. Photogramm. Remote Sens. 126, 24–37. doi: 10.1016/j.isprsjprs.2017.02.001 38826717

[B35] Kheir A. M. S. Govind A. Nangia V. Devkota M. Elnashar A. Omar M. E. D. . (2024). Developing automated machine learning approach for fast and robust crop yield prediction using a fusion of remote sensing, soil, and weather dataset. Environ. Res. Commun. 6, 041005. doi: 10.1088/2515-7620/ad2d02

[B36] Lausch A. Zacharias S. Dierke C. Pause M. Kühn I. Doktor D. . (2013). Analysis of vegetation and soil patterns using hyperspectral remote sensing, EMI, and gamma-ray measurements. Vadose Zone J. 12, 1–15. doi: 10.2136/vzj2012.0217

[B37] LeDell E. Poirier S. (2020). H2O AutoML: Scalable automatic machine learning. arXiv preprint arXiv:2006.02582

[B38] Li X. He B. Ding K. Guo W. Huang B. Wu L. (2022). Wide-area and real-time object search system of UAV. Remote Sens. 14, 1234. doi: 10.3390/rs14051234 30654563

[B39] Li Z. Zhou X. Cheng Q. Zhai W. Mao B. Li Y. . (2023). An integrated feature selection approach to high water stress yield prediction. Front. Plant Sci. 14, 1289692. doi: 10.3389/fpls.2023.1289692 38111876 PMC10726204

[B40] Lingwal S. Bhatia K. K. Singh M. (2024). A novel machine learning approach for rice yield estimation. J. Exp. Theor. Artif. Intell. 36, 337–356. doi: 10.1080/0952813X.2022.2062458 37339054

[B41] Ministry of Agriculture of the People’s Republic of China(MOA) (2008). Methods for Measurement and Acceptance of National High-Yield Grain Creation (Trial) (Beijing: Ministry of Agriculture).

[B42] Motohka T. Nasahara K. N. Oguma H. Tsuchida S. (2010). Applicability of green-red vegetation index for remote sensing of vegetation phenology. Remote Sens. 2, 2369–2381. doi: 10.3390/rs2102369 30654563

[B43] Nicodemus K. K. Malley J. D. Strobl C. Ziegler A. (2010). The behaviour of random forest permutation-based variable importance measures under predictor correlation. BMC Bioinf. 11, 110. doi: 10.1186/1471-2105-11-110 20187966 PMC2848005

[B44] Pearson K. (1895). X. Contributions to the mathematical theory of evolution.—II. Skew variation in homogeneous material. Philos. Trans. R. Soc. London. (A.) 186, 343–414. doi: 10.1098/rsta.1895.0010 34341189

[B45] Prokhorenkova L. Gusev G. Vorobev A. Dorogush A. V. Gulin A. (2018). CatBoost: unbiased boosting with categorical features. In: Advances in Neural Information Processing Systems 31 (NeurIPS 2018), (Red Hook, NY: Curran Associates, Inc.), 6638–6648. doi: 10.48550/arXiv.1706.09516

[B46] Reyniers M. Walvoort D. J. J. De Baardemaaker J. (2006). A linear model to predict with a multi‐spectral radiometer the amount of nitrogen in winter wheat. Int. J. Remote Sens. 27, 4159–4179. doi: 10.1080/01431160600791650 37339054

[B47] Rondeaux G. Steven M. Baret F. (1996). Optimization of soil-adjusted vegetation indices. Remote Sens. Environ. 55, 95–107. doi: 10.1016/0034-4257(95)00186-7

[B48] Roujean J.-L. Breon F.-M. (1995). Estimating PAR absorbed by vegetation from bidirectional reflectance measurements. Remote Sens. Environ. 51, 375–384. doi: 10.1016/0034-4257(94)00114-3

[B49] Rouse J. W. Haas R. H. Schell J. A. Deering D. W. (1974). Monitoring vegetation systems in the Great Plains with ERTS. In: Third ERTS-1 Symposium NASA (Washington, DC: NASA), 309–317.

[B50] Shapley L. S. (1953). Quota solutions of n-person games. In: Contributions to the Theory of Games, Volume II, Kuhn H. W. Tucker A. W. editors. (Princeton, NJ: Princeton University Press), 343–360.

[B51] Snoek J. Larochelle H. Adams R. P. (2012). “ Practical bayesian optimization of machine learning algorithms”, in: Proceedings of the 26th International Conference on Neural Information Processing Systems - Volume 2 (Red Hook, NY: Curran Associates, Inc.), 2951–2959. doi: 10.48550/arXiv.1206.2944

[B52] Song Q. Zhang G. Zhu X.-G. (2013). Optimal crop canopy architecture to maximise canopy photosynthetic CO2 uptake under elevated CO2 - a theoretical study using a mechanistic model of canopy photosynthesis. Funct. Plant Biol. 40, 108–124. doi: 10.1071/FP12056 32481092

[B53] Sulik J. J. Long D. S. (2016). Spectral considerations for modeling yield of canola. Remote Sens. Environ. 184, 161–174. doi: 10.1016/j.rse.2016.06.016 38826717

[B54] Swain K. P. Mohapatra S. K. Sahoo S. K. (2025). Enhancing predictive modeling across industries with automated machine learning: applications in insurance and agriculture. Discover Sustainability 6, 167. doi: 10.1007/s43621-025-00965-9 30311153

[B55] Tang B. Zhou J. Zhao C. Pan Y. Lu Y. Liu C. . (2025). Using UAV-based multispectral images and CGS-YOLO algorithm to distinguish maize seeding from weed. Artif. Intell. Agric. 15, 162–181. doi: 10.1016/j.aiia.2025.02.007 38826717

[B56] Tukey J. W. (1977). Exploratory Data Analysis (Reading, Mass: Addison-Wesley Pub. Co).

[B57] van Klompenburg T. Kassahun A. Catal C. (2020). Crop yield prediction using machine learning: a systematic literature review. Comput. Electron. Agric. 177, 105709. doi: 10.1016/j.compag.2020.105709 38826717

[B58] Wan L. Cen H. Zhu J. Zhang J. Zhu Y. Sun D. . (2020). Grain yield prediction of rice using multi-temporal UAV-based RGB and multispectral images and model transfer – a case study of small farmlands in the South of China. Agric. For. Meteorol. 291, 108096. doi: 10.1016/j.agrformet.2020.108096 38826717

[B59] Wang Z. Tan X. Ma Y. Liu T. He L. Yang F. . (2024). Combining canopy spectral reflectance and RGB images to estimate leaf chlorophyll content and grain yield in rice. Comput. Electron. Agric. 221, 108975. doi: 10.1016/j.compag.2024.108975 38826717

[B60] Weiss M. Jacob F. Duveiller G. (2020). Remote sensing for agricultural applications: a meta-review. Remote Sens. Environ. 236, 111402. doi: 10.1016/j.rse.2019.111402 38826717

[B61] Whitmire C. D. Vance J. M. Rasheed H. K. Missaoui A. Rasheed K. M. Maier F. W. (2021). Using machine learning and feature selection for alfalfa yield prediction. AI 2, 71–88. doi: 10.3390/ai2010006 30654563

[B62] Yuan W. Meng Y. Li Y. Ji Z. Kong Q. Gao R. . (2023). Research on rice leaf area index estimation based on fusion of texture and spectral information. Comput. Electron. Agric. 211, 108016. doi: 10.1016/j.compag.2023.108016 38826717

[B63] Zhang Q. Cheng Y.-B. Lyapustin A. I. Wang Y. Zhang X. Suyker A. . (2015). Estimation of crop gross primary production (GPP): II. Do scaled MODIS vegetation indices improve performance? Agric. For. Meteorol. 200, 1–8. doi: 10.1016/j.agrformet.2014.09.003 38826717

[B64] Zhang C. Harrison P. A. Pan X. Li H. Sargent I. Atkinson P. M. (2020). Scale sequence joint deep learning (SS-JDL) for land use and land cover classification. Remote Sens. Environ. 237, 111593. doi: 10.1016/j.rse.2019.111593 38826717

[B65] Zhang J. Qiu X. Wu Y. Zhu Y. Cao Q. Liu X. . (2021). Combining texture, color, and vegetation indices from fixed-wing UAS imagery to estimate wheat growth parameters using multivariate regression methods. Comput. Electron. Agric. 185, 106138. doi: 10.1016/j.compag.2021.106138 38826717

[B66] Zhao C. He D. Qiao Y. (2013). Identification method of multi-feature weed based on multi-spectral images and data mining. Trans. Chin. Soc Agric. Eng.(Trans. CSAE) 29, 192–198.

[B67] Zheng H. Ma J. Zhou M. Li D. Yao X. Cao W. . (2020). Enhancing the nitrogen signals of rice canopies across critical growth stages through the integration of textural and spectral information from unmanned aerial vehicle (UAV) multispectral imagery. Remote Sens. 12, 957. doi: 10.3390/rs12060957 30654563

[B68] Zhou H. Huang F. Lou W. Gu Q. Ye Z. Hu H. . (2025). Yield prediction through UAV-based multispectral imaging and deep learning in rice breeding trials. Agric. Syst. 223, 104214. doi: 10.1016/j.agsy.2024.104214 38826717

[B69] Zhou X. Zheng H. B. Xu X. Q. He J. Y. Ge X. K. Yao X. . (2017). Predicting grain yield in rice using multi-temporal vegetation indices from UAV-based multispectral and digital imagery. ISPRS J. Photogramm. Remote Sens. 130, 246–255. doi: 10.1016/j.isprsjprs.2017.05.003 38826717

