# Supplementary material for: Rice yield prediction using UAV-based multispectral imagery and AutoGluon across regions and field scales
Source: Front Plant Sci. 2026 Jul 8;17:1866530. doi: 10.3389/fpls.2026.1866530 (PMC13388542; doi:10.3389/fpls.2026.1866530)
Supplement: Supplementary file 1 [file SupplementaryFile1.docx]

Rice yield prediction using UAV-based multispectral imagery and AutoGluon across regions and field scales

# 1 Appendix S. Supplementary data:

| 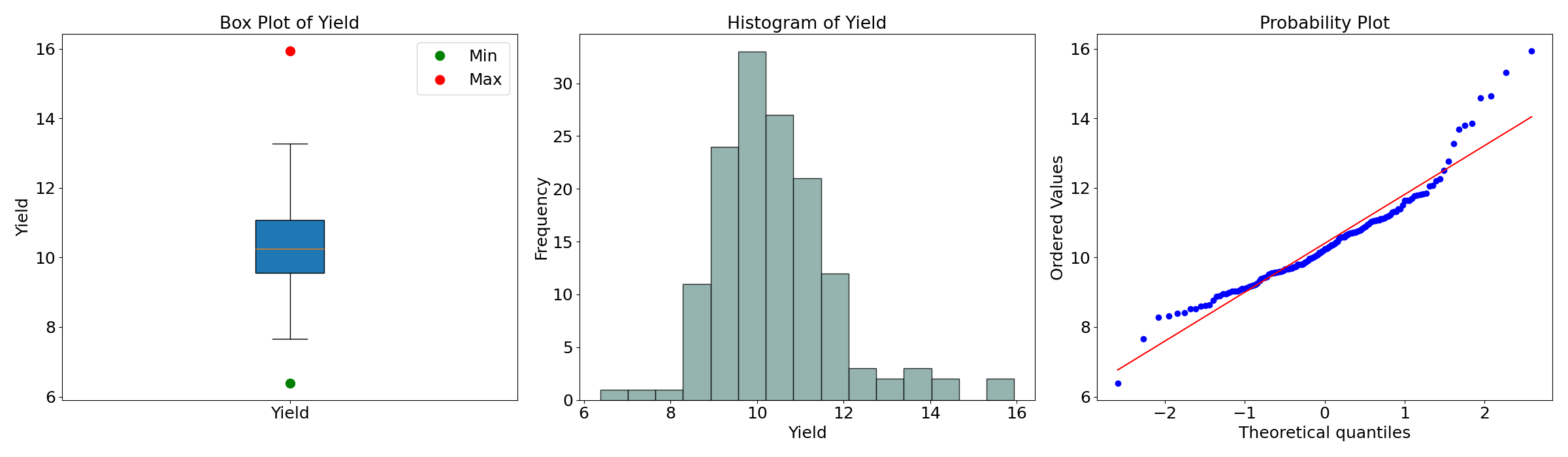  Figure S.1. Histogram of yield showing the frequency distribution of sample yields. |
| --- |

| 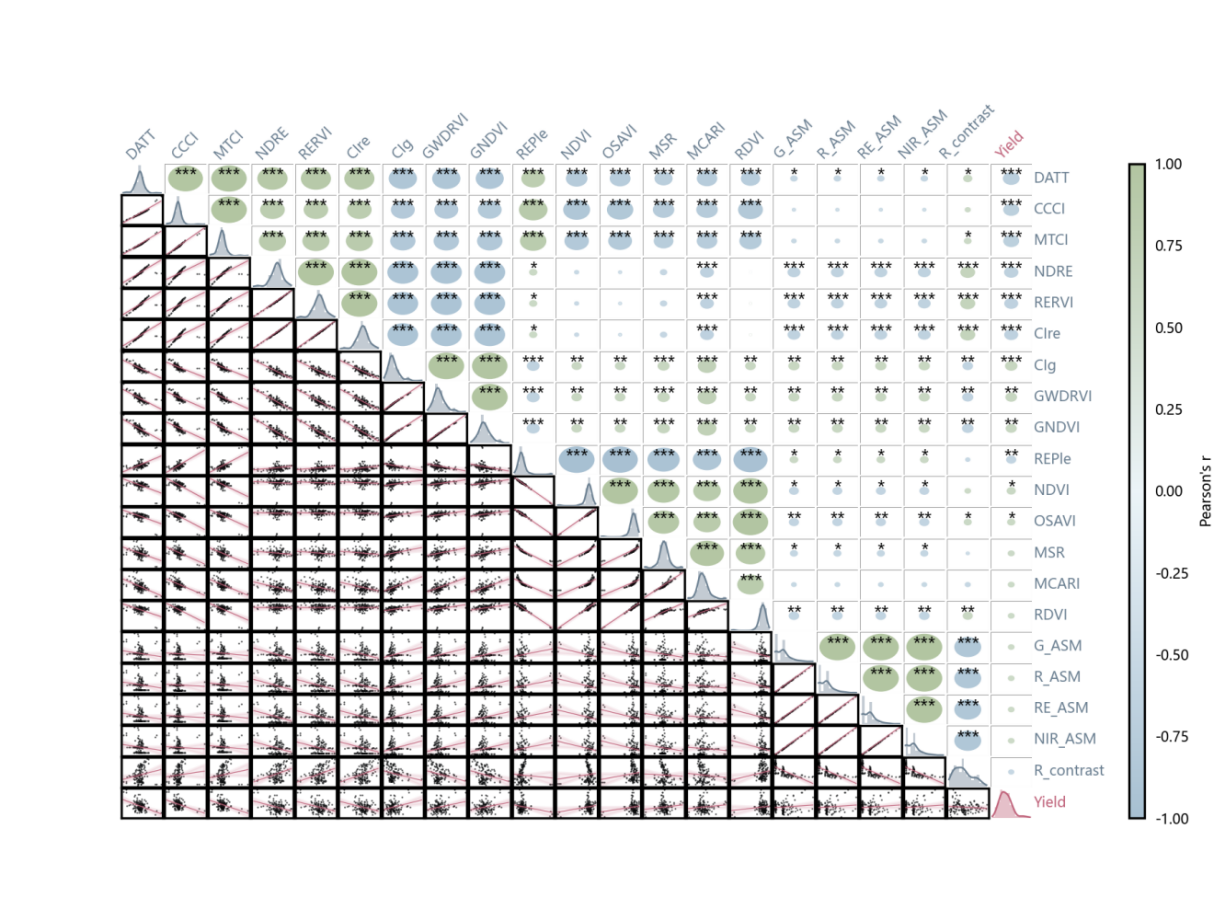  Figure S.2. Correlation matrix among remote sensing features and their linear correlation analysis with rice yield. Asterisks (*, **, ***) denote significance levels at 0.1, 0.01, and 0.001, respectively. |
| --- |
|  |

Table S.1

Technical specifications of the UAV.

| Project Name | Technical Parameter |
| --- | --- |
| Bare machine weight (with blades and RTK module) | 951 g |
| Maximum horizontal flight speed | 15 meters per second (normal gear) Forward flight: 21 meters per second, side flight: 20 meters per second, rear flight: 19 meters per second (sport gear) |
|  |  |
| Maximum flight time (windless environment) | 43 minutes |
| Maximum hovering time (windless environment) | 37 minutes |
| Maximum driving range | 32 kilometers |
| GNSS | GPS+Galileo+BeiDou+GLONASS(GLONASS is only supported when the RTK module is enabled) |
|  |  |
| Multispectral camera image sensor | 1/2.8-inch CMOS, with 5 million effective pixels |
| Multispectral camera band | Green：560nm±16nm；Red：650nm±16nm；Rededge：730nm±16nm；NIR：860nm±26nm |
|  |  |
| Maximum photo size of multispectral camera | 2592*1944 |
| Multispectral camera photo format | TIFF |
| Pan-tilt stabilization system | Three-axis mechanical pan-tilt (pitch, roll, translation) |
| Type of perception system | Omnidirectional binocular vision system, supplemented by infrared sensors at the bottom of the fuselage |
| RTK position accuracy | RTK fixed solution: Horizontal: 1cm+1ppm; Vertical: 1.5cm+1ppm |
|  |  |

Table S.2

Feature ranking based on Pearson correlation coefficients.

| Feature screening method | Feature | Correlation |
| --- | --- | --- |
| Pearson correlation coefficient | DATT | -0.421422 |
|  | CCCI | -0.397772 |
|  | MTCI | -0.396565 |
|  | NDRE | -0.347070 |
|  | RERVI | -0.336059 |
|  | CIre | -0.336059 |
|  | CIg | 0.289549 |
|  | GWDRVI | 0.266616 |
|  | GNDVI | 0.251631 |
|  | REPle | -0.221374 |
|  | NDVI | 0.193244 |
|  | OSAVI | 0.165359 |
|  | MSR | 0.129255 |
|  | MCARI | 0.123818 |
|  | RDVI | 0.121841 |
|  | G_ASM | 0.117967 |
|  | R_ASM | 0.117765 |
|  | RE_ASM | 0.117764 |
|  | NIR_ASM | 0.117760 |
|  | R_contrast | -0.100963 |
|  | RE_contrast | -0.100658 |
|  | VIopt | 0.099844 |
|  | G_variance | -0.085633 |
|  | R_variance | -0.079852 |
|  | GRVI | 0.076887 |
|  | RE_dissimilarity | -0.075543 |
|  | NIR_contrast | -0.072350 |
|  | G_entropy | -0.068139 |
|  | R_entropy | -0.063435 |
|  | R_dissimilarity | -0.060897 |
|  | NIR_entropy | -0.060876 |
|  | RE_entropy | -0.060487 |
|  | EVI | -0.060410 |
|  | RE_mean | 0.060262 |
|  | MTVI2 | 0.057314 |
|  | NIR_dissimilarity | -0.051598 |
|  | NIR_mean | 0.047589 |
|  | G_dissimilarity | -0.041051 |
|  | R_mean | 0.037564 |
|  | NIR_variance | -0.027841 |
|  | TVI | 0.024636 |
|  | R_correlation | 0.021660 |
|  | G_homogeneity | 0.021398 |
|  | RE_homogeneity | 0.018065 |
|  | G_energy | 0.016645 |
|  | NIR_homogeneity | 0.016246 |
|  | R_energy | 0.015808 |
|  | RE_energy | 0.015292 |
|  | NIR_energy | 0.015264 |
|  | R_homogeneity | 0.012558 |
|  | G_mean | -0.011864 |
|  | G_correlation | -0.008937 |
|  | NIR_correlation | -0.007700 |
|  | G_contrast | -0.007553 |
|  | RE_variance | 0.004839 |
|  | RE_correlation | -0.002167 |

Table S.3

Feature ranking based on Random Forest (RF) feature importance.

| Feature screening method | Feature | Importance |
| --- | --- | --- |
| The importance of RF characteristics | NIR_variance | 0.129463 |
|  | G_contrast | 0.044613 |
|  | R_variance | 0.043801 |
|  | RERVI | 0.043796 |
|  | RE_variance | 0.040830 |
|  | CIg | 0.036071 |
|  | RE_contrast | 0.035852 |
|  | DATT | 0.034903 |
|  | GWDRVI | 0.031348 |
|  | CCCI | 0.025187 |
|  | NDRE | 0.025162 |
|  | GRVI | 0.023871 |
|  | MTCI | 0.023838 |
|  | CIre | 0.022075 |
|  | G_variance | 0.020742 |
|  | EVI | 0.019762 |
|  | R_contrast | 0.019530 |
|  | RE_dissimilarity | 0.017233 |
|  | GNDVI | 0.016689 |
|  | G_mean | 0.016450 |
|  | RE_mean | 0.014530 |
|  | REPle | 0.013494 |
|  | NIR_contrast | 0.012911 |
|  | NIR_entropy | 0.012714 |
|  | NIR_homogeneity | 0.012360 |
|  | R_ASM | 0.012302 |
|  | MTVI2 | 0.012073 |
|  | MSR | 0.011729 |
|  | OSAVI | 0.011509 |
|  | RE_entropy | 0.011239 |
|  | G_dissimilarity | 0.010877 |
|  | G_correlation | 0.010754 |
|  | NIR_dissimilarity | 0.010694 |
|  | NIR_mean | 0.010391 |
|  | R_mean | 0.010223 |
|  | MCARI | 0.009815 |
|  | VIopt | 0.009810 |
|  | R_entropy | 0.009003 |
|  | TVI | 0.008541 |
|  | R_dissimilarity | 0.008135 |
|  | NIR_energy | 0.007954 |
|  | RDVI | 0.007903 |
|  | NDVI | 0.007695 |
|  | RE_ASM | 0.007579 |
|  | NIR_ASM | 0.007553 |
|  | R_correlation | 0.007449 |
|  | G_energy | 0.007155 |
|  | RE_homogeneity | 0.007012 |
|  | G_homogeneity | 0.006981 |
|  | RE_correlation | 0.006977 |
|  | G_entropy | 0.006235 |
|  | NIR_correlation | 0.006006 |
|  | R_energy | 0.005614 |
|  | R_homogeneity | 0.005419 |
|  | G_ASM | 0.005362 |
|  | RE_energy | 0.002786 |

Table S.4

Feature ranking based on AutoGluon feature importance.

| Feature screening method | Feature | Importance |
| --- | --- | --- |
| The importance of AutoGluon features | NIR_variance | 0.053324 |
|  | RE_variance | 0.051153 |
|  | R_variance | 0.044906 |
|  | NIR_dissimilarity | 0.039395 |
|  | G_variance | 0.033238 |
|  | R_contrast | 0.031773 |
|  | DATT | 0.030799 |
|  | CCCI | 0.025204 |
|  | GWDRVI | 0.024026 |
|  | R_homogeneity | 0.023726 |
|  | R_dissimilarity | 0.023643 |
|  | R_correlation | 0.023200 |
|  | RE_contrast | 0.022931 |
|  | MTVI2 | 0.021955 |
|  | G_contrast | 0.021699 |
|  | G_entropy | 0.021617 |
|  | G_correlation | 0.021357 |
|  | CIg | 0.021140 |
|  | G_homogeneity | 0.020818 |
|  | RE_dissimilarity | 0.020447 |
|  | RE_mean | 0.020331 |
|  | NIR_mean | 0.019928 |
|  | MTCI | 0.019788 |
|  | GNDVI | 0.019660 |
|  | R_mean | 0.019530 |
|  | G_dissimilarity | 0.019425 |
|  | GRVI | 0.019373 |
|  | G_mean | 0.018503 |
|  | NIR_entropy | 0.018303 |
|  | CIre | 0.017827 |
|  | TVI | 0.017112 |
|  | NIR_contrast | 0.016986 |
|  | RE_entropy | 0.016611 |
|  | RE_correlation | 0.016141 |
|  | G_ASM | 0.015599 |
|  | RE_energy | 0.015170 |
|  | RE_ASM | 0.014879 |
|  | NDRE | 0.014817 |
|  | EVI | 0.014014 |
|  | MCARI | 0.013957 |
|  | RDVI | 0.013679 |
|  | VIopt | 0.013567 |
|  | NIR_energy | 0.013451 |
|  | R_entropy | 0.012749 |
|  | NIR_homogeneity | 0.012249 |
|  | G_energy | 0.011632 |
|  | RE_homogeneity | 0.011469 |
|  | REPle | 0.011400 |
|  | RERVI | 0.011325 |
|  | R_energy | 0.009324 |
|  | R_ASM | 0.009093 |
|  | NIR_correlation | 0.008844 |
|  | NIR_ASM | 0.008695 |
|  | OSAVI | 0.008219 |
|  | MSR | 0.008205 |
|  | NDVI | 0.007276 |
